# Immunogenetics of Idiopathic Inflammatory Myopathies: The Role of HLA Genes Within and Beyond the Ancestral Haplotype

**DOI:** 10.3390/genes17050517

**Published:** 2026-04-27

**Authors:** Olga Gumkowska-Sroka, Kacper Kotyla, Przemysław Kotyla

**Affiliations:** 1 Department of Rheumatology and Clinical Immunology, Medical University of Silesia, Voivodeship Hospital No. 5, 41-200 Sosnowiec, Poland; oag@poczta.onet.pl; 2 Chair of Internal Medicine, Rheumatology and Clinical Immunology, Medical University of Silesia, 40-055 Katowice, Poland; 3 Department of Medical Biophysics, Medical University of Silesia Katowice, 40-752 Katowice, Poland; kacper.kotyla@gmail.com

**Keywords:** idiopathic inflammatory myopathies, ancestral haplotype, GWAS

## Abstract

Idiopathic inflammatory myopathies constitute a group of immune-mediated disorders primarily affecting skeletal muscle, but they may also lead to significant involvement of internal organs. These conditions are highly heterogeneous, encompassing diverse clinical manifestations and multiple underlying pathophysiological mechanisms. A unifying feature across this disease spectrum is an autoimmune response characterized by the production of highly specific autoantibodies, which are detected in the majority of patients. Genetic studies have identified the principal susceptibility background as the 8.1 ancestral haplotype within the HLA region on chromosome 6. However, genetic predisposition extends beyond HLA loci and includes numerous genes encoding key molecules involved in cytokine production, the regulation of immune signaling pathways, and metabolic processes. In this paper, we review the currently identified genetic loci associated with inflammatory myopathies, with particular emphasis on the HLA system, as well as non-HLA genes and newly identified candidates.

## 1. Introduction

Idiopathic inflammatory myopathies (IIMs), which are among the prototypical representatives of connective tissue diseases, constitute a group of rare autoimmune disorders. Their incidence is estimated to range from 0.2 to 2 cases per 100,000 person-years, while prevalence varies between 2 and 25 cases per 100,000 individuals, depending on geographic location, the population studied, and the statistical methods employed [1,2]. Moreover, epidemiological data clearly indicate that the prevalence of idiopathic inflammatory myopathies is higher in females and increases with advancing age [3,4].

The hallmark clinical feature of IIMs is muscle involvement, characterized by varying degrees of inflammatory activity, muscle weakness, and impaired mobility [5]. However, these conditions are systemic in nature and frequently present with extramuscular manifestations, including cutaneous rash, interstitial lung disease, polyarthritis, and an increased risk of malignancy [6].

Idiopathic inflammatory myopathies typically present with symmetric proximal muscle weakness, leading to impaired ambulation and difficulties with activities such as climbing stairs, rising from a seated position, lifting objects, and performing overhead tasks. Myalgia is a common feature and often precedes the onset of clinically overt disease, with the exception of patients with inclusion body myositis. Weakness of the neck extensors may result in dropped head syndrome. Involvement of other muscle groups can give rise to clinical manifestations such as camptocormia, dysphagia, and dysphonia, which may represent presenting symptoms and can occur across different subtypes of myositis [7,8,9]. Oropharyngeal muscle weakness frequently leads to dysphagia [10], a common complication with an estimated prevalence of approximately 36% among patients with myositis and up to 56% in those with inclusion body myositis. Dysphagia contributes significantly to weight loss and reduced quality of life and may result in life-threatening complications such as aspiration pneumonia.

The pattern and rate of muscle involvement vary according to the myopathy subtype. In patients with dermatomyositis and antisynthetase syndrome, disease onset typically evolves over weeks to months, whereas in immune-mediated necrotising myopathy, progression may be more rapid [11,12]. Depending on the specific subtype of myopathy, a range of additional signs and symptoms may be present. These include characteristic cutaneous manifestations in dermatomyositis, predominant distal muscle involvement in inclusion body myositis, and prominent arthritis in antisynthetase syndrome. Such variability suggests that, despite being grouped under the common umbrella of idiopathic inflammatory myopathies, these conditions differ substantially in their underlying pathophysiological mechanisms, genetic background, histopathological features, clinical course, and response to treatment [6].

Over the past five decades, there have been substantial advances in our understanding of this group of muscle disorders. Early classification was largely based on the system proposed by Bohan and Peter, who identified five principal subtypes of inflammatory myopathies. These included polymyositis, defined as a primary inflammatory disorder of skeletal muscle, and dermatomyositis, described as polymyositis accompanied by characteristic cutaneous involvement. In addition, they recognized three further categories associated with specific clinical contexts: myositis occurring in conjunction with other connective tissue diseases (overlap syndromes), myositis associated with malignancy, and juvenile dermatomyositis/polymyositis, often linked to vasculitic features [13,14].

Idiopathic inflammatory myopathies are heterogeneous in nature. They differ substantially in terms of clinical presentation, underlying pathophysiology, and the mechanisms of autoimmune response involved. This heterogeneity is clearly reflected in the diverse autoantibody profiles serological spectrum of autoantibodies associated with specific myopathy subtypes, indicating that distinct autoimmune mechanisms may underlie the initiation and propagation of the muscle inflammation [15].

Over recent decades, the conceptual framework of inflammatory myopathies has evolved in parallel with the recognition of distinct clinical entities within this spectrum of disorders. Newly defined conditions, such as antisynthetase syndrome and immune-mediated necrotising myopathy, have been identified, and additional subgroups within previously established entities—particularly dermatomyositis—have also been proposed [6,16].

Currently, the most widely accepted subclassification of idiopathic inflammatory myopathies in adults comprises six major clinical entities: dermatomyositis, inclusion body myositis, immune-mediated necrotising myopathy, antisynthetase syndrome, overlap myositis, and polymyositis [17]. However, this classification remains the subject of ongoing scientific debate, as certain categories—particularly polymyositis—are poorly defined and intrinsically heterogeneous. Polymyositis lacks specific autoantibodies, a characteristic clinical phenotype, and a distinctive histopathological pattern, which has led to questions regarding its validity as a discrete entity [18,19].

Additional controversy surrounds the classification of cancer-associated myositis. It remains unclear whether it should be regarded as an independent clinical entity or rather as a manifestation of an immune response triggered by an underlying neoplastic process.

Idiopathic inflammatory myopathies are classified according to the EULAR/ACR (European Alliance of Associations for Rheumatology/American College of Rheumatology) criteria; however, several criteria exist also in regard to particular subgroups of the diseases [20].

The wide range of clinical presentations and the heterogeneity of pathophysiological backgrounds suggest that multiple genetic mechanisms may contribute to the initiation and progression of the inflammatory process in patients with IIM.

This review aims to examine key immunogenetic mechanisms underlying idiopathic inflammatory myopathies (both adults and juvenile), with particular focus on HLA genes, especially those constituting the 8.1 ancestral haplotype. In addition, we consider selected genetic variants outside the HLA system that influence both innate and adaptive immune responses.

We also highlight polymorphisms in genes involved in immune cell function, cytokine production, and inflammatory signaling, as well as their associations with specific myopathy subtypes and autoantibody production.

### Methodology

A systematic literature search was conducted to identify English-language, peer-reviewed original articles and review papers published between January 2010 and April 2026. The search was performed across the PubMed, MEDLINE, ScienceDirect, Scopus, and ResearchGate databases. The search strategy employed combinations of standardized medical subject headings (MeSH) and relevant keywords, including “idiopathic inflammatory myopathy,” “dermatomyositis,” “polymyositis,” “antisynthetase syndrome,” “inclusion body myositis,” and “immune-mediated necrotizing myopathy,” in conjunction with terms such as “genome-wide association studies” “GWAS,” “genes,” “genetic regulation,” “ImmunoChip,” and “single nucleotide polymorphism.” Additionally, review articles identified through the search were screened, and their reference lists were examined to ensure the inclusion of any potentially relevant studies not captured in the initial search. The Authors screened the titles and abstracts identified through the comprehensive search and selected studies based on predefined inclusion and exclusion criteria. We excluded from the review conference reports, commentaries, theses, and non-peer-reviewed publications. Any discrepancies or uncertainties were resolved through discussion among the investigators. Where overlapping data from the same author appeared in multiple publications—despite differences in format or title—the data were included only once. We included cross-sectional, case–control, and cohort studies. Studies involving non-human subjects, as well as, case reports, editorials, and commentaries, were excluded from this systematic review and meta-analysis.

Two authors evaluated the quality of each study. Each item was rated as “Yes,” “No,” “Unclear,” or “Not applicable”. Based on these scores, studies were categorized as having a high risk of bias (low quality), moderate risk of bias (moderate quality), or low risk of bias (high quality).

We initially identified 2637 individual records. The majority were duplicates, arising from overlapping MeSH terms across different databases as well as within individual databases. After removal of duplicates, 236 records remained and were further assessed for compliance with the search strategy criteria. Ultimately, 62 papers met the preliminary inclusion criteria, of which 35 were original research articles subsequently included and cited in the manuscript.

## 2. Major Histocompatibility Complex

Genome-wide association studies (GWASs) of single-nucleotide polymorphisms (SNPs) in individuals of European ancestry with dermatomyositis or polymyositis have identified the strongest disease associations within the major histocompatibility complex (MHC) region on chromosome 6. This region, one of the most complex in the human genome, encompasses genes encoding proteins that regulate immune responses. The development of the ImmunoChip platform has further enhanced the ability to detect and refine these HLA-associated genetic signals [21].

Major histocompatibility complex (MHC) molecules, which are fundamentally involved in the recognition of antigens and autoantigens, remain a central focus of scientific investigation [22]. Detailed analyses have demonstrated that multiple HLA genes are closely associated with a range of autoimmune diseases, including diabetes mellitus, myasthenia gravis, vitiligo, and connective tissue disorders [23]. The 8.1 ancestral haplotype is extremely long (more than four megabases) conserved set of genes located at chromosome 6. It consists from a combination of HLA-A1*, CCw7, *B8*, TNFAB*a2b3, TNFN*S TNFN*S, C2*C, Bf*s, C4A*Q0, C4B*1, DRB1*03:01, DRB3*01:01, DQA1*05:01, DQB1*02:01 alleles [24]. It is a highly conserved haplotype, typically inherited as a single block from a common ancestor; consequently, its structure remains relatively stable and is minimally affected by recombination across generations [25].

**Figure 1:**
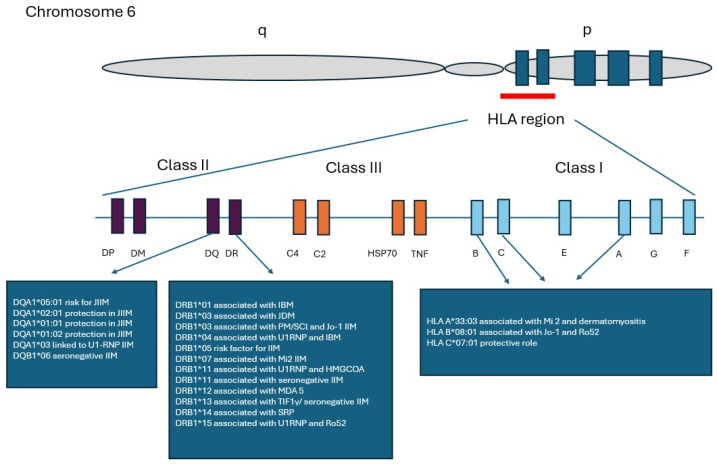
HLA region with genes involved in inflammatory myopathies.

Thus, the strongest genetic predisposition to idiopathic inflammatory myopathies is localized within the major histocompatibility complex (MHC), which harbors specific human leukocyte antigen (HLA) alleles that confer susceptibility to these disorders [26]. Among the various genetic contributors, the 8.1 ancestral haplotype (AH) represents a key factor associated with susceptibility to idiopathic inflammatory myopathies. The largest genetic study in idiopathic inflammatory myopathies, conducted by the Myositis Genetics Consortium, analyzed 2566 patients from 14 countries and demonstrated the strongest disease associations with alleles of the 8.1 ancestral haplotype (*8.1 AH-HLA A1-B8-DR3-DQ2*), specifically HLA-DRB1*03:01 in polymyositis and HLA-B*08:01 in dermatomyositis (Figure 1) [27,28].

The role of the 8.1 ancestral haplotype is likely not confined to a single disease subtype, as associations with its alleles have been observed across multiple forms of idiopathic inflammatory myopathies. These include subgroups defined by distinct autoantibody profiles, such as anti-Jo-1, anti-PM/Scl, and anti-cN1A autoantibodies [29]. But even in this subpopulation of the patients seropositivity for Anti-Jo-1 autoantibodies, an additional independent association has been identified with the class I HLA-B*08:01 [30] and class II DRB1*03 [29]. This is not surprising as HLA-B*08:01 is also a component of the 8.1 ancestral haplotype. The genetic contribution to idiopathic inflammatory myopathies varies and is influenced by the degree of familial relatedness within the studied population. For example, in European populations, heritability has been estimated at approximately 22–24% among first-degree relatives and siblings [31].

Anti-TIF-1 autoantibodies were directed against a 155 kDa nuclear protein identified as a risk factor for the development of cancer-associated myositis (CAM) [32]. In one study, an association between Anti-TIF1 autoantibodies and the 8.1 ancestral haplotype was demonstrated; however, this relationship was observed only in patients with juvenile-onset disease and not in those with adult-onset myositis [33,34,35].

These findings suggest that, despite the established link between TIF1-γ and CAM, distinct genetic backgrounds may underlie juvenile- and adult-onset forms of myositis, supporting the notion that they represent biologically different conditions. In line with this, somatic mutations and loss of heterozygosity in the *TRIM33* gene, which encodes TIF1-γ, have been reported in tumors from adults with cancer-associated myositis. In contrast, in juvenile myositis, environmental factors—such as infections—are thought to play a more prominent role in disease pathogenesis [36]. Quite recently somatic mutations and loss of heterozygosity in autoantibody-related genes as tripartite motif containing 33, tripartite motif containing 66, *MORC* family CW-type zinc finger 3 (*MORC3*), *Chromodomain Helicase DNA Binding Protein 4*, and *IFIH1*, interferon-induced helicase C domain-containing protein 1—corresponding to known myositis-specific autoantibodies have been identified [37].

The development of myositis-specific autoantibodies is also strongly influenced by genetic factors. For example, data from Caucasian populations indicate that HLA-DRB1*03:01 is associated with the presence of Anti-Jo-1 and Anti-PL-12 antibodies [38]. In contrast, in African American populations, Anti-Mi-2 antibodies have been linked to HLA-DRB1*03:02, highlighting population-specific genetic associations [39].

The 8.1 ancestral haplotype is not uniformly distributed across all racial groups. Although it is highly prevalent in Caucasian populations, it occurs significantly less frequently in African American and Asian populations. Correspondingly, the association between the “typical” haplotype and idiopathic inflammatory myopathies (IIMs), commonly observed in Caucasians, is rare in Asian populations.

In contrast, in Asian cohorts—particularly among Korean patients—the typical association of IIM with the 8.1 ancestral haplotype is replaced by a different genetic pattern. Specifically, the haplotype HLA-DRB1*12:02, which is rare in Caucasians, is associated with seropositivity for anti-MDA5 autoantibodies in Korean patients.

The main shared feature between Caucasian and Korean patients with IIM appears to be the presence of HLA-DRB1*07:01 and seropositivity for anti-Mi-2 antibodies.

These findings highlight that a diverse immunogenetic background underlies and regulates the autoimmune response in IIM.

Moreover, HLA-DRB1*0101 and *0405 have been shown to be associated with the presence of anti-MDA-5 antibodies in patients with dermatomyositis within the Japanese population [40]. Additionally, the signal transducer and activator of transcription 4 (STAT4) gene, a critical mediator in immune regulatory signaling pathways, is pathophysiologically implicated in the initiation and progression of severe autoimmune conditions [41]. STAT4 functions as a transcription factor that promotes the polarization of T helper cells toward Th1 and Th17 responses and is considered a genetic risk factor for the development of idiopathic inflammatory myopathies [42].

Myositis-specific autoantibodies, a group of autoantibodies closely linked to the development specific subtypes of myopathies (MSAs), are tightly associated with genetic factors as follows:

1. HLA-DRB1*12:02 associated with anti-MDA5,
2. HLA-DRB1*14:03 with anti-SRP,
3. HLA-DRB1*07:01 with anti-Mi-2,
4. HLA-DRB1*13:01 with anti-TIF1γ
5. HLA-DRB1*11:01 with anti-HMGCR antibody
6. HLA-DRB1*01:01, and HLADRB1*04:10 and inclusion body myositis in Japanese patients (IBM) [43,44,45,46].
7. HLA-DRB1*08:03 and anti-aminoacyl–tRNA synthetase in Korean patients
8. HLA-DRB1*14:03 and anti-signal recognition particles in Korean patients
9. HLA-DPB1*17:01 and anti-Mi2 in Korean patients.

Human leukocyte antigen (HLA) genetic variability plays a critical role in the pathogenesis of numerous autoimmune diseases. HLA molecules are central to antigen recognition by T cells and are essential for processes such as T-cell receptor development, maintenance of peripheral tolerance, and immune responses to environmental stimuli. It is well established that geographic location and ethnicity can influence susceptibility to autoimmune disorders. For example, the HLA-DRB1*03:01 and HLA-DQA1*05:01 alleles have been identified as risk factors for myositis in Western populations [47,48]. Contrary to this PM susceptibility in Japanese population is linked to the presence of DRB1*08:03 [49] and frequencies of DRB1*01:01, DRB1*04:10, and DRB1*15:02 were high in the Japanese patients with IBM [46].

In a transracial gene-mapping study involving White, African American, Mexican American, and Japanese patients with idiopathic inflammatory myopathies, myositis-specific autoantibodies were found to be similarly distributed across racial groups. However, the frequencies of the HLA–DRB1*03:01 (DR3), DQA1*05:01, and DQB1*02:01 (DQ2) alleles were significantly increased in White patients, particularly those with polymyositis and, most prominently, in patients seropositive for myositis-specific autoantibodies. In other ethnic groups, excluding the Japanese cohort, only the frequencies of HLA–DQA1*05:01 and the structurally related DQA1*04:01 alleles were elevated [48]. Subsequently, Shamim et al. described the association of different HLA DRB1* and DQA1* with anti-Mi-2 antibodies in Mexican, Guatemalan and North American Caucasian patients with idiopathic inflammatory myopathy [50].

Another study examining HLA–autoantibody associations was conducted in the Korean population. The findings indicate a distinct immunogenetic background in Korean patients with myositis compared to Caucasian populations. Differences were also observed in the prevalence of specific autoantibodies. In this study, the most common autoantibody was Anti-MDA5, which demonstrated a strong association with HLA-DRB1*12:02 [43]. In a recent observational case–control study, Jassal et al. evaluated 147 biopsy-confirmed patients with idiopathic inflammatory myopathies and 114 ethnically matched healthy controls. The authors performed high-resolution HLA genotyping and conducted a comparative analysis of amino acid frequencies between the two groups [51].

According to the findings of this study, HLA-C*07:01 was associated with a protective effect in idiopathic inflammatory myopathies. Further analyses demonstrated an association between HLA-A*33:03 and dermatomyositis, whereas HLA-DQB1*06:03 was linked to polymyositis. Patients seropositive for anti-Mi-2 antibodies exhibited strong associations with HLA-A*33:03, HLA-DQA1*02:01, and HLA-DQB1*03:03. In contrast, seropositivity for anti-Ro52 antibodies was associated with HLA-B*08:01 [51].

## 3. Molecular Aspects of Idiopathic Inflammatory Myopathies

The Genetics Scientific Interest Group (MYOGEN), affiliated with the International Myositis Assessment & Clinical Studies Group, is a scientific consortium focused on elucidating the genetic basis of idiopathic inflammatory myopathies (IIMs). In 2015, MYOGEN completed a genome-wide association study in IIM using whole-genome genotyping data. This was followed in 2016 by a larger study employing the ImmunoChip, designed to evaluate 186 loci previously implicated in 12 autoimmune diseases. Both the GWAS and ImmunoChip studies analyzed genotyped single-nucleotide polymorphism (SNP) data from patients with adult or juvenile dermatomyositis (DM) and polymyositis (PM), alongside geographically matched controls, with data integrated across the various IIM subtypes [28,52].

The largest genetic association study conducted to date in idiopathic inflammatory myopathies, using the ImmunoChip, included 2566 patients with polymyositis, dermatomyositis, juvenile dermatomyositis, and inclusion body myositis [29]. The study demonstrated that both the HLA region and the *PTPN22* gene are associated with idiopathic inflammatory myopathies at genome-wide significance. Additionally, nine loci showed suggestive associations, including *UBE2L3*, *CD28*, and *TRAF6*.

Subgroup analyses revealed distinct genetic differences among polymyositis, dermatomyositis, and juvenile dermatomyositis. Notably, *PTPN22* was associated at genome-wide significance with PM (rs2476601), but not with DM or JDM, indicating that this genetic effect may be specific to PM. Further suggestive associations were identified, including *IL18R1* and *RGS1* in PM, and *GSDMB* in DM.

HLA imputation analyses confirmed that the alleles HLA-DRB1*03:01 and HLA-B*08:01, which form part of the 8.1 ancestral haplotype, are the strongest genetic factors associated with susceptibility to idiopathic inflammatory myopathies, consistent with previous reports. Of particular interest is the finding regarding the *STAT4* gene, whose role has also been confirmed in the Japanese population [53]. According to the study results, the lead single-nucleotide polymorphism in this region (rs4853540) was associated with a protective effect. Additionally, an independent risk association was identified for rs10174238, along with a further potential independent effect at rs932169 [27].

In a large European cross-disease shared autoimmunity meta-analysis evaluating 6.5 million single-nucleotide polymorphisms across 11,678 patients with seropositive immune-mediated inflammatory diseases, including systemic sclerosis, systemic lupus erythematosus, idiopathic inflammatory myopathies, and rheumatoid arthritis, and 19,704 unaffected controls of European ancestry, the authors identified 26 genome-wide significant loci shared across these conditions, highlighting common genetic contributors to autoimmunity.

Among these, five novel independent loci—*NAB1*, *KPNA4-ARL14*, *DGKQ*, *LIMK1*, and *PRR12*—had not been previously associated with these diseases. Notably, these loci are implicated in key immunological and cellular processes, including interferon signaling, epidermal growth factor–mediated pathways, response to methotrexate treatment, cytoskeletal organization, and coagulation [54]. These findings substantiate the existence of a shared genetic architecture across autoimmune diseases, including idiopathic inflammatory myopathies (IIMs), irrespective of their underlying etiology, distinct genetic predispositions, or clinical phenotypes, even among well-characterized connective tissue disorders.

## 4. Genetic and Clinical Associations by Serologically Defined IIM Subgroups

The presence of autoantibodies in idiopathic inflammatory myopathies is nearly universal, whereas seronegative cases, although reported in large patient cohorts, remain exceptional. Furthermore, autoantibodies—classified into myositis-specific and myositis-associated antibodies—not only facilitate diagnosis but also enable the classification of these disorders into well-defined clinical subgroups, and may reflect different disease mechanisms driven by genetic variation within HLA alleles. Although muscle weakness, inflammation, and tissue damage are common clinical features, idiopathic inflammatory myopathies (IIMs) are best conceptualized as a spectrum of disorders rather than precisely defined clinical entities. Serological markers that facilitate the classification of IIMs into more specific clinical subgroups are influenced by genetic factors. These include genes within the human leukocyte antigen (HLA) complex, as well as non-HLA genes regulating key immune mechanisms, such as cytokine production, inflammatory signaling pathways, and antigen presentation. Consequently, in addition to genes with established roles in IIMs, several novel genetic loci have been associated with distinct subtypes of these condition [26,27,55]. Accordingly, studies have aimed to investigate the relationship between autoantibody profiles and clinical manifestations in the context of HLA-DRB1*, HLA-DQA1*, and HLA-DQB1* allele expression. Based on the predominant autoantibody profile, eight distinct autoantibody-defined subgroups were identified [29]. In 2019, Rothwell et al. conducted a study involving 2582 Caucasian patients with idiopathic inflammatory myopathies (IIMs), stratified into 12 subgroups based on autoantibody seropositivity. The authors reported associations with eight autoantibodies that reached genome-wide significance (*p* < 5 × 10^−8^). The strongest associations with the 8.1 ancestral haplotype were observed for anti-Jo-1 (HLA-B*08:01, HLA-DRB1*03:01), anti-PM/Scl (HLA-DQB1*02:01), and anti-cN1A autoantibodies (HLA-DRB1*03:01). Associations independent of this haplotype were identified for anti-Mi-2 (HLA-DRB1*07:01, *p* = 4.92 × 10^−13^) and anti-HMGCR autoantibodies (HLA-DRB1*11, *p* = 5.09 × 10^−6^). Furthermore, a strong HLA association was observed in patients with anti-TIF1 autoantibodies, particularly with HLA-DQB1*02:01 and HLA-DQB1*02:02 [55].

### 4.1. Subgroup 1: Anti-Ro52-Dominated

Autoantibodies directed against Ro52 (TRIM21) are frequently detected in several connective tissue diseases. In conjunction with disease-specific autoantibodies, they are classified as myositis-associated antibodies and are also observed in conditions such as systemic lupus erythematosus, systemic sclerosis, idiopathic inflammatory myopathies, and rheumatoid arthritis [56,57]. Associations between anti-Ro52 antibodies and HLA alleles, particularly HLA-DRB1*03 and HLA-DRB1*15, have been reported; however, these associations did not remain significant after correction for multiple testing.

### 4.2. Subgroup 2: Anti-U1RNP-Dominated

Anti-U1RNP antibodies are widely recognized as a serological hallmark of mixed connective tissue disease (MCTD), but they may also be detected in other connective tissue diseases, including systemic lupus erythematosus, idiopathic inflammatory myopathies, systemic sclerosis and primary Sjögren’s syndrome, where they can significantly influence the clinical phenotype [58,59,60,61,62].

Patients with idiopathic inflammatory myopathies who are positive for anti-U1RNP antibodies tend to present at a younger age and exhibit less prominent initial muscle involvement. This subgroup is characterized by a marked female predominance, symmetric proximal muscle weakness, and a necrotizing pattern on muscle biopsy [56].

In this population, increased frequencies of the HLA-DRB1*04, HLA-DRB1*11, HLA-DRB1*15, HLA-DQA1*03, and HLA-DQB1*03 alleles have been observed. In contrast, no signals with class I amino acid were detected after correction for multiple comparisons. Further analysis demonstrated that the association with HLA-DRB1*15 was independent of the signals observed for HLA-DRB1*04, HLA-DRB1*11, HLA-DQA1*03, and HLA-DQB1*03. The similar conclusions come from Polish study on MCTD where association with HLA-DRB1*15 allele has been observed [63].

### 4.3. Subgroup 3: Anti-PM/Scl-Dominated

Anti-PM/Scl antibodies are typically observed in patients with scleromyositis, a condition representing an overlap between systemic sclerosis and inflammatory myopathy [64,65]. In this subgroup, significant associations have been reported with HLA class II alleles, particularly HLA-DRB1*03, HLA-DQA1*05, and HLA-DQB1*02.

### 4.4. Subgroup 4: Anti-Mi2-Dominated

Patients with anti-Mi-2β antibodies typically present with a classic dermatomyositis phenotype, characterized by prominent cutaneous manifestations, severe muscle weakness, and elevated baseline creatine kinase levels [66]. In agreement with this, in the cohort studied, individuals positive for anti-Mi-2 antibodies predominantly exhibit a dermatomyositis clinical phenotype. Within this subgroup, the frequencies of the HLA-DRB1*07 and HLA-DQA1*02 alleles are higher compared with other patient subgroups.

### 4.5. Subgroup 5: Anti-Jo1-Dominated

Anti-Jo-1 antibodies (also known as anti-histidyl tRNA synthetase antibodies) are currently recognized as a serological hallmark of idiopathic inflammatory myopathies, as reflected in the EULAR/ACR classification criteria [20]. Among the aminoacyl–tRNA synthetase autoantibodies, anti-Jo-1 is the most frequently detected, and its presence is strongly associated with a distinct clinical subtype of IIM known as antisynthetase syndrome (ASyS) [12,67].

ASyS is now recognized as a separate subgroup within the IIM spectrum and is characterized by the development of interstitial lung disease, inflammatory myopathy, and arthritis, often accompanied by characteristic features such as “mechanic’s hands” and Raynaud’s phenomenon [68].

In this patient subgroup, increased frequencies of HLA class II alleles, particularly HLA-DRB1*03, have been observed, along with specific amino acid associations, including arginine at position 74 of *HLA-DRB1* and aspartic acid at position 9 of HLA-B.

### 4.6. Subgroup 6: Anti-Jo1/Ro52-Dominated

This subgroup represents an excellent example of the coexistence of myositis-specific and myositis-associated autoantibodies, which together exert a substantial influence on the resulting clinical phenotype. As expected, all patients within this subgroup were classified as having antisynthetase syndrome and demonstrated a markedly higher prevalence of Raynaud’s phenomenon. Strong associations have been observed between this phenotype and HLA alleles HLA-DRB1*03, HLA-DQA1*05, and HLA-DQB1*02.

### 4.7. Subgroup 7: Anti-TIF1γ-Dominated

Anti-transcription intermediary factor 1 gamma (anti-TIF1γ) antibodies, a subset of myositis-specific autoantibodies, are commonly associated with an increased risk of malignancy [37]. This relationship is typically referred to as cancer-associated myositis (CAM). Malignancy occurs relatively frequently in this context, reaching approximately 13% according to data from the Euromyositis registry [69].

Most cases of CAM present as adult-onset dermatomyositis, with an incidence approximately twofold higher than that observed in polymyositis (3.8–7.7 vs. 1.7–2.2) [70]. Accordingly, in the evaluated cohort, the majority of patients seropositive for anti-TIF1γ antibodies were clinically classified as cases of dermatomyositis. This anti-TIF1γ-positive phenotype is characterized by significant associations with HLA class II alleles, including HLA-DRB1*07, HLA-DRB1*01, HLA-DQA1*02, and *HLA-DQB1*. At present, the precise immunogenetic mechanisms underlying cancer-associated myopathy (CAM) and the genetic regulation of this phenomenon remain largely unknown. It is also unclear whether this genotype confers an increased risk of cancer development or promotes a cancer-induced immune response with cross-reactivity targeting the skin and muscle

### 4.8. Subgroup 8: Negative for Analyzed Autoantibodies

Idiopathic inflammatory myopathies are currently recognized as autoimmune diseases that are closely associated with myositis-specific autoantibodies, which not only facilitate diagnosis but are also widely used for precise disease classification. This is particularly relevant for polymyositis, which is not consistently associated with any specific myositis-specific autoantibodies; consequently, the validity of this subgroup is increasingly questioned.

Therefore, the absence of detectable myositis-specific autoantibodies should be interpreted with caution, as it may not accurately reflect the true serological status but rather result from the lack of testing for newly identified antibodies. Additionally, a subtype of immune-mediated necrotizing myopathy (IMNM) lacking seropositivity for anti-HMGCR and anti-SRP antibodies is recognized as a truly seronegative form. In the study by Leclair et al., seropositivity for anti-HMGCR and anti-SRP antibodies was not assessed; therefore, patients with IMNM may have been misclassified within the “seronegative” category [29]. Moreover, in patients with immune-mediated necrotizing myopathy who are seropositive for anti-HMGCR antibodies, the HLA class II allele HLA-DRB1*11:01 is present in more than 70% of cases [71]. More recent findings indicate that seronegative subgroups exhibit higher frequencies of HLA-DRB1*13, HLA-DQA1*01, and HLA-DQB1*06, which may reflect distinct immunological mechanisms and genetic predisposition within this patient population.

The results suggest that subgrouping IIM based on autoantibody profiles and HLA variants may reflect distinct pathogenetic mechanisms better than subgrouping based on traditional clinical/histopathological classifications.

### 4.9. Juvenile Idiopathic Inflammatory Myopathies (JIIMs)

Juvenile idiopathic inflammatory myopathies (JIIMs) are a heterogeneous group of rare connective tissue diseases affecting children and adolescents. These conditions primarily involve striated muscles but may also affect the skin and internal organs, including the lungs, gastrointestinal tract, joints, heart, and central nervous system. Juvenile dermatomyositis is the most common form of juvenile idiopathic inflammatory myopathies (JIIMs), accounting for approximately 80% of cases, followed by antisynthetase syndrome (ASyS), immune-mediated necrotizing myopathy (IMNM), overlap syndromes, and amyopathic myositis [72].

With the similarities to adult-onset myositis, a range of myositis-specific autoantibodies associated with distinct clinical has been identified. Moreover, these autoantibodies, in conjunction with the clinical presentation, can be used to classify juvenile idiopathic inflammatory myopathies into clinically relevant subtypes. Notably, however, the frequencies of specific autoantibodies differ substantially compared with those observed in adult populations [73]. The principal pathophysiological hallmark of juvenile idiopathic inflammatory myopathies is vasculopathy accompanied by endothelial damage, which distinguishes these conditions from adult-onset myopathies, in which skeletal muscle tissue represents the primary target of disease involvement [70].

Key haplotypes linked to JDM risk include DRB1*03:01, DQA1*05:01, and HLA-B*08, while protective haplotypes including DQA1*02:01, DQA1*01:01, and DQA1*01:02 have been identified.

Similar to adult-onset inflammatory myopathies, juvenile idiopathic inflammatory myopathies are closely associated with the 8.1 ancestral haplotype of the major histocompatibility complex [28]. In contrast to adult-onset disease, the allele HLA-DRB1*03:01 and amino acid position 37 within *HLA-DRB1* confer risk factors for JIIM [74]. However, when patients with juvenile dermatomyositis were analyzed separately, key haplotypes associated with this subgroup were identified. Risk haplotypes included HLA-DRB1*03:01, HLA-DQA1*05:01, and HLA-B*08, which are associated with increased susceptibility to the disease, whereas haplotypes included HLA-DQA1*02:01, HLA-DQA1*01:01, and HLA-DQA1*01:02 have the protective effect [44]. Additional differences between juvenile- and adult-onset idiopathic inflammatory myopathies have been observed in patients seropositive for anti-HMGCR antibodies. In juvenile cases, anti-HMGCR seropositivity is associated with HLA-DRB1*07:01, whereas in adult-onset disease it is more commonly linked to HLA-DRB1*11:01 [75]. Other important genetic contributors in this field include loci outside the major histocompatibility complex, such as *PTPN22* and the rs2304256 variant in TYK2, which have been identified as risk factors for the development of both juvenile and adult inflammatory myopathies [76,77].

A substantial body of evidence supports the role of major histocompatibility complex genes in the development of idiopathic inflammatory myopathies. These findings were recently reinforced by a study conducted by Zhu et al. [78] who performed association analyses in a cohort of 14,903 individuals (3206 patients and 11,697 controls). In addition, the authors identified several novel risk loci and susceptibility genes, including *FCRLA*, *NFKB1*, *IRF4*, *DCAKD*, and *ATXN2* in overall IIM, *NEMP2* in polymyositis, *ACBC11* in dermatomyositis, and *PSD3* in patients with myositis seropositive for Anti-Jo-1 antibodies [79].

Recently, Jassal et al. identified HLA associations linked to clinically and serologically defined subgroups of idiopathic inflammatory myopathies, enabling the identification of potentially immunogenic epitopes. Their findings demonstrated a direct relationship between HLA-mediated genetic susceptibility and distinct clinical and autoantibody-defined subgroups of IIM.

The analysis across the studied cohort revealed a protective effect associated with the HLA-C*07:01 allele. Furthermore, specific HLA alleles were found to correlate with particular disease subtypes, including HLA-A*33:03 with dermatomyositis and HLA-DQB1*06:03 with polymyositis. The Anti-Mi-2 subgroup showed strong associations with HLA-A*33:03, HLA-DQA1*02:01, and HLA-DQB1*03:03. Finally, Anti-Ro52 positivity was associated with the HLA-B*08:01 haplotype [51].

## 5. Genes Outside HLA System

Several genes involved in the regulation of immune responses, cytokine synthesis, and the transmission of inflammatory signals have recently been proposed as novel risk factors for the development of idiopathic inflammatory myopathies. Among these, *NR1H4* (FXR) has attracted considerable attention, particularly in dermatomyositis [79].

Another top candidate in JDM, *TXNIP*, interacts with the nucleotide-binding oligomerization domain (NOD)-like receptor protein 3 (NLRP3) inflammasome, which has been suggested to associate with myositis

A significant role for genes outside the HLA region—particularly those involved in the regulation of both innate and adaptive immune responses—has been substantiated by ImmunoChip studies. These include *STAT4* (Signal Transducer and Activator of Transcription 4), *TRAF6* (TNF Receptor-Associated Factor 6), *UBE2L3* (Ubiquitin-Conjugating Enzyme E2 L3), and *PTPN22* (Protein Tyrosine Phosphatase Non-Receptor Type 22), all of which have been implicated in the pathogenesis of idiopathic inflammatory myopathies. The *PTPN22* variant rs2476601 demonstrated genome-wide significance in patients with idiopathic inflammatory myopathies, particularly in those exhibiting a polymyositis clinical phenotype [78]. The roles of *PTPN22* and *STAT4* in the regulation of immune responses are of considerable importance, as both genes are key modulators of signaling pathways mediated through the T cell receptor (TCR) and B cell receptor [80]. In parallel, *UBE2L3* (also known as UBcH7), a member of the E2 ubiquitin-conjugating enzyme family, regulates multiple signalling pathways, including NF-κB, GSK3β/p65, and DNA double-strand break repair pathways. It is therefore recognized as a biologically active factor involved in the pathophysiological mechanisms underlying several autoimmune diseases [81]. In a large cross-sectional, case–control study from the UK involving patients with various subtypes of IIM, Chinoy et al. reported an association of *PTPN22 C1858T* with idiopathic inflammatory myopathy in White Individuals [76]. In addition, *PTPN22 1858T* was not associated with dermatomyositis in a GWAS of patients with adult or juvenile dermatomyositis, as shown in Table 1 [82].

Studies aimed at identifying novel genes and genetic variants, particularly those involved in the regulation of immune responses, are currently ongoing. Recent findings have highlighted the potential roles of genes such as *SDK2*, *LINC00924*, *NAB1*, *GSDMB*, and *CCL21*, which have been associated with several subtypes of idiopathic inflammatory myopathies—Table 2.

Although their precise contributions to disease development and progression remain to be fully elucidated, functional analyses suggest that genetic variability affecting these genes may lead to the dysregulation of immune responses, thereby contributing to disease pathogenesis.

The genetic regulation of pathological processes in IIM extends far beyond effector factors such as cytokines and receptors. Recent genome-wide studies focusing on non-HLA genes have identified several novel loci with suggestive genome-wide significance involved in the regulation of immune and metabolic processes that may contribute to the pathogenesis of idiopathic inflammatory myopathies (Table 3).

Among these, the *Sidekick* (*SDK*) gene has recently emerged as a potential susceptibility factor for IIM. Originally described in Drosophila, SDK encodes the Sidekick protein, one of the largest members of the immunoglobulin superfamily [86]. Within the immune system, SDK proteins are thought to function as antigen-binding molecules, cytokine receptors, and recognition molecules that facilitate interactions between immunocompetent cells, including communication across distinct immune cell populations [87]. However, the primary physiological function of this molecule is related to the development and maintenance of nervous tissue and neural circuits in humans [88]. Another gene identified in the study, *LINC00924*—a long non-coding RNA (lncRNA)—has been extensively investigated as a biomarker in cancer development [89]. Consequently, findings related to this gene in IIMs represent novel insights that suggest a potential overlap between carcinogenesis and autoimmunity. The role of *LINC00924* was recently confirmed by Rothwell et al., who demonstrated a genome-wide significant association for this gene in idiopathic inflammatory myopathies [84].

Of the various genetic loci identified in their analysis, the *NAB1* gene—a corepressor induced by type I interferons—warrants particular attention, given the pivotal role of interferon-mediated pathways in several autoimmune diseases, including IIMs [90]. These findings highlight the prognostic roles of the lncRNA *LINC00924* and *NAB1* in idiopathic inflammatory myopathies, while also underscoring the importance of regulatory pathways and cell-to-cell interactions as key elements of IIM pathogenesis. Other findings in the study point to the role of *GSDMB*, which encodes gasdermins—a family of proteins involved in pore-forming activity in cell membranes during inflammatory cell death (pyroptosis) [91]. Additionally, the *CCL21* gene encodes the chemokine CCL21, which is expressed in the endothelium of lymphoid tissues, lymphatic vessels, and stromal cells of the spleen and appendix. *CCL21* regulates the trafficking of dendritic cells, NK cells, and T and B lymphocytes to sites of inflammation [92]. Another key factor in this field is *CCR5* (chemokine receptor 5), which demonstrated suggestive genome-wide significance in the study by Rothwell et al. [84]. Given its role as a G protein–coupled receptor expressed on T cells and macrophages, *CCR5* is integral to immune responses and may therefore be pathophysiologically involved in the development of idiopathic inflammatory myopathies. Among the others genes identified in the study by Rothwell et al. [84], the *TEC* gene is of particular interest. *TEC* encodes a key non-receptor tyrosine kinase primarily involved in intracellular signaling pathways that govern immune cell development, activation, and differentiation. Accordingly, it plays a significant role in both immune and autoimmune responses [93]. The autoimmune nature of idiopathic inflammatory myopathies (IIMs) is further supported by the evaluation of *LTβR* gene. *LTβR* (*TNFRSF3*) is a cell-surface receptor that plays a critical role in lymphoid organogenesis, immune cell homeostasis, and the organization of peripheral lymphoid tissues. It regulates the homeostasis of neutrophils, NK cells, and iNKT cells, contributes to IgA production, and is involved in the control of chronic inflammation, liver regeneration, and oncogenic processes [94]. Collectively, these functions suggest that *LTβR* is implicated in autoimmune responses characteristic of IIM, as previously demonstrated [84]. The clinical significance of these findings requires further evaluation; however, they point to the involvement of subtle and precise mechanisms of autoimmunity and inflammation that are closely linked to the nature of idiopathic inflammatory myopathies.

## 6. Conclusions

The 8.1 ancestral haplotype remains a key genetic component in susceptibility to inflammatory myopathies, which is unsurprising given that the genes comprising this haplotype have been consistently associated with multiple autoimmune disorders across numerous genetic studies. The role of the HLA system in general, and the 8.1 ancestral haplotype in particular, has been established in multiple studies and has been further corroborated by more recent investigations [51,95,96,97]. However, recent findings increasingly highlight the importance of additional components of the immune system, including cytokines, chemokines, and critical molecules involved in immune signaling pathways.

These advances contribute to a more comprehensive understanding of immune system dysfunction in the myositis spectrum and may facilitate the development of novel therapeutic strategies targeting key elements of the pathophysiological milieu. Importantly, given the substantial heterogeneity among currently recognized subtypes of inflammatory myopathies, such therapeutic approaches may need to be tailored to the specific constellation of genes underlying the pathogenesis of each subtype.

Several important advances have been achieved in recent years. Efforts to link specific serotypes in idiopathic inflammatory myopathies (IIMs), particularly those associated with myositis-specific antibodies (MSAs) and myositis-associated antibodies (MAA), have broadened our understanding of the disease spectrum [98,99]. These developments underscore the need for future classifications of IIM that integrate serological profiles with clinical and histopathological features, on one hand, and gene expression patterns, on the other hand. This integrated approach may be particularly relevant in light of emerging therapeutic observations suggesting the need for more targeted treatments beyond corticosteroid administration alone.

The establishment of serologically defined subsets of IIM, closely linked to genetic predisposition and regulation, enables the construction of genetically informed clinical phenotypes, especially in cases where two or more autoantibodies are present in a single patient [29,51]. The results suggest that subgrouping IIM based on autoantibody profiles and HLA variants may reflect distinct pathogenetic mechanisms better than subgrouping based on traditional clinical/histopathological classifications. This is particularly important when combinations of MSA and MAA are involved. A notable example is anti-Ro52 seropositivity, which is clinically associated with several autoimmune disorders, including lupus, systemic sclerosis, Sjögren’s syndrome, and IIM [100].

It is plausible that the co-expression of multiple antibodies results from the activation of distinct autoantigen-processing pathways. Consequently, the clinical phenotype may be modified by the presence of additional antibodies, which in turn may have implications for therapeutic decision-making. Moreover, somatic mutations and loss of heterozygosity in genes associated with autoantibody production may directly contribute to the synthesis of specific autoantibodies, thereby providing a direct link between genotype and the immune status of patients [37].

These findings have not yet been translated into precision medicine or targeted therapeutic approaches; however, advances in this field are paving the way toward more precise and individualized treatment strategies.

## Figures and Tables

**Table 1:** Classic non-HLA susceptibility genes in myositis.

Gene	SNP	Function	Role in IIM	Reference
*BLK*	rs2736340 rs7812879 rs13277113 rs17799348	B cel receptor signalling kinase	Association with PM/DM in Chinse population especially with ILD and Japanese population Suggestive genome significance	[49,79,83]
*STAT4*	rs4853540 rs7574865 T rs4853540	Transcription factor in Il12/23 signalling	Genome-wide significance in IIM associated with IIM and in Japanese population	[49,53,84]
*PTPN22*	rs2476601	Tyrosine phosphatase regulating T cell activation	Associated with IBM Associated with IMM independent of 8.1AH, suggestive genome significance	[79,84,85]
*UBE2L3*	rs11089637	NFκB signalling	Associated with IIM overlap with SLE, RA	[84]
*HCP5*	rs3132090		Genome-wide association with Jo-1 seropositivity	[79]

ILD, interstitial lung disease; PM, polymyositis; DM, dermatomyositis; IIM, idiopathic inflammatory myopathy; SLE, systemic lupus erythematosus; RA, rheumatoid arthritis. 8.1 AH ancestral haplotype. NFκB signalling nuclear factor κB.

**Table 2:** Newly discovered non-HLA loci (recent GWAS/meta-analysis).

Gene	SNP	Biologic Function/Name	Role in IIM	Reference
*SDK2*	rs7209879		genome-wide significance in IIM	[84]
*LINC00924*	rs8040452	long intergenic non-protein coding RNA 924	genome-wide significance in IIM	
*FCRLA*	rs6668534	Fc receptor like A	Protective role in IIM genome wide significance	[79]
*STAT4*	rs4853540	Targets interferon regulatory motif	Protective role in IIM	[79]
*ATXN2*	rs35350651	Ataxin-2	Protective role in IMM Genome-wide significance for PM	[79]
*DCAKD*	rs9898793 rs12950988		High probability for IMNM	[79]
*NFKB1*	rs230514	Nuclear factor κ B	Increased risk for IIM genome wide significance	[79]
*IRF4*	Rs12203592	Transcription factor controlling B cell differentiation	Increased risk for IIM Genome-wide associations with DM and JDM	[79]
*ABCB 11*	rs145940036	Encoding bile salt export pump	Increased risk for DM	[79]
*PINX1*	rs113538396		Genome-wide associations with PM	
*NEMP2*	rs 74925618	encodes a nuclear envelope integral membrane protein 2	Increased risk for PM	[79]
*NAB 1*	rs6733720	NGFI-A binding protein 1	genome-wide significance in IIM	[84]
*PSD3*	rs6991531	Pleckstrin And Sec 7 Domain Containing 3	Genome-wide associations with anti-Jo-1 seropositivity	[79]

According to the literature, a *p*-value of <5 × 10^−8^ is considered indicative of genome-wide significance. NGFI-A Nerve Growth Factor-Inducible protein A.

**Table 3:** Recently identified GWAS loci with suggestive significance in IIM.

Gene	SMP	Name	Role in IIM	Reference
*PHTF1-PTPN22*	rs6679677		Suggestive significance in PM	[84]
*DGKQ*	rs6599390	Diacylglycerol Kinase Theta	IIM	[84]
*TEC*	rs80105690	Tec Protein Tyrosine Kinase	IIM	[84]
*PLCL1*	rs1518359	Luarhythmo/Phytoclock 1	IIM	[84]
*LTBR*	rs11064180	Lymphotoxin Beta Receptor	IIM	[84]
*CCR5*	Rs41490645	C-C motif chemokine receptor 5	Suggestive genome significance in IBM	[84]

Genes with suggestive significance in IIM (*p* < 2.25 × 10^−5^). IIM, idiopathic inflammatory myopathy; PM, polymyositis; IBM, inclusion body myositis. *PHTF1-PTPN22*—Putative Homeodomain Transcription Factor 1-Protein Tyrosine Phosphatase Non-Receptor Type 22.

## Data Availability

No new data were created or analyzed in this study. Data sharing is not applicable to this article.
